# Alignment of biological networks by integer linear programming: virus-host protein-protein interaction networks

**DOI:** 10.1186/s12859-020-03733-w

**Published:** 2020-11-18

**Authors:** Mercè Llabrés, Gabriel Riera, Francesc Rosselló, Gabriel Valiente

**Affiliations:** 1 Department of Mathematics and Computer Science, University of the Balearic Islands, Palma de Mallorca, E-07122 Spain; 2 Balearic Islands Health Research Institute, Palma de Mallorca, E-07010 Spain; 3 Algorithms, Bioinformatics, Complexity and Formal Methods Research Group, Technical University of Catalonia, Barcelona, E-08034 Spain

**Keywords:** Systems biology, Virus-host protein-protein interaction, Integer linear programming, Network alignment, Graph matching

## Abstract

**Background:**

The alignment of protein-protein interaction networks was recently formulated as an integer quadratic programming problem, along with a linearization that can be solved by integer linear programming software tools. However, the resulting integer linear program has a huge number of variables and constraints, rendering it of no practical use.

**Results:**

We present a compact integer linear programming reformulation of the protein-protein interaction network alignment problem, which can be solved using state-of-the-art mathematical modeling and integer linear programming software tools, along with empirical results showing that small biological networks, such as virus-host protein-protein interaction networks, can be aligned in a reasonable amount of time on a personal computer and the resulting alignments are structurally coherent and biologically meaningful.

**Conclusions:**

The implementation of the integer linear programming reformulation using current mathematical modeling and integer linear programming software tools provided biologically meaningful alignments of virus-host protein-protein interaction networks.

## Background

Many meaningful questions in molecular biology have been successfully answered through their translation into alignment problems for different mathematical structures. From simple structures, such as genomic or proteomic sequences, to richer structures, such as complex networks or whole biological systems, pairwise and multiple alignment have been used to compare these structures, inferring features and new biological relations from their alignment.

Several methods and software tools have been already introduced for the alignment of biological networks, including protein-protein interaction networks, metabolic pathways, and gene regulatory networks. They are addressed to solve interesting biological questions, such as the inference of protein-protein interactions and protein functions, the regulation of biological processes, and the metabolic capabilities of microorganisms. The alignment and analysis of protein-protein interaction networks has become a key ingredient to obtain functional orthologs and discover protein-protein interactions and their associated functions, as well as evolutionary conserved assembly pathways of protein complexes.

In the general network setting, and hence also in the particular case of protein-protein interaction networks, an alignment between two networks is an injective, but possibly partial, mapping from the set of nodes in one network (the source network) to the set of nodes in the other network (the target network). When the mapping defining the alignment has as domain the whole set of nodes of the source network, the alignment becomes an embedding of the source into the target network. Since biological networks are large networks, with hundreds to thousands of nodes and edges, most of the techniques developed for their alignment [1–5] are heuristic, and the alignments obtained by applying these techniques to the same biological networks often differ considerably and do not provide a true, consensus alignment. On the other hand, an exact solution to the network alignment problem can be obtained by an integer quadratic programming formulation [6], but its linearization [7] has a huge number of binary variables and constraints.

In this paper, we present a compact integer linear programming reformulation of the protein-protein interaction network alignment problem, which can be solved using state-of-the-art mathematical modeling and integer linear programming software tools. We also present empirical results showing that small biological networks, such as the virus-host protein-protein interaction networks in the STRING Viruses database [8], can be aligned in a reasonable amount of time on a personal computer and the resulting alignments are structurally coherent and biologically meaningful.

## Results

The STRING Viruses database [8] contains sequences for 9,660,620 viral and host proteins and protein-protein interaction data for 230 viruses and 3 hosts: *Homo sapiens* (11,437,065 interactions), *Saccharomyces cerevisiae* (2,007,278 interactions), and *Escherichia coli* (1,166,900 interactions). We downloaded from STRING Viruses the virus-host protein-protein interaction data for *Homo sapiens* and all the protein sequence data (see Availability of data and materials).

Each of the protein-protein interactions is annotated with a combined score, an indicator of confidence ranging from 0 to 1, where a combined score of 0.5 indicates that roughly every second interaction might be a false positive. Therefore, we discarded any protein-protein interaction with a combined score under 0.510, keeping only interactions in the last 10% of the distribution of combined scores. Also, host-host protein-protein interactions were discarded since the alignment purpose in this experiment is the relation between the proteins of a virus and its host. Nevertheless, in a more general setting host-host protein-protein interactions can be also considered. Further, we discarded the smallest networks, those with 64 or less interactions, and focused our alignment experiments on the remaining 25 largest networks, which have between 56 and 735 viral and host proteins and between 65 and 957 virus-host protein-protein interactions. These networks are listed in Table S1, ranked by the number of interactions, and the viral proteins involved in them are listed in Tables S2–S26 [see Additional file 1].

Then, we aligned all possible pairs of these 25 networks. Due to the symmetry of the network alignment problem, we actually aligned 25·24/2=300 pairs of networks. We performed each of these 300 alignments using the compact integer linear programming formulation presented in this paper with AMPL version 2018.10.22 [9] and Gurobi Optimizer version 8.1.0, and also with some of the most popular protein-protein interaction network alignment tools: PINALOG [1], SPINAL [2], HubAlign [3], L-GRAAL [4], and AligNet [5], using default parameters for all of them. All of the alignments where computed using a personal computer with an Intel Core i7-8550U quad-core processor at 1.80 GHz and 32 GB of memory running Ubuntu 18.04 LTS. We took either the optimal solution or the best feasible solution that could be computed within a solver time limit of 60 minutes.

While our method is aimed at finding exact solutions to the problem of aligning protein-protein interaction networks, all of the aforementioned protein-protein interaction network alignment tools use an heuristic algorithm to obtain the final alignment. The general idea behind all of these alignment tools, is to define a node similarity measure that combine the similarity of the protein sequences with some network structure similarity. Then, the actual alignment is obtained based on node similarity. More precisely, in PINALOG, network structures are “communities,” which are scored and aligned based on a node similarity score that combines protein sequence similarity and GO terms, and aligned communities are extended to obtain the network alignment. In SPINAL, node similarity score is defined based on sequence similarity of nodes and of their neighbours, this score is iterated until some stability is reached, and the network alignment is obtained by a greedy, seed-and-extend approach. In HubAlign, network structures are “hubs” and “bottlenecks,” a score or weight is assigned to each node and edge of a network using an iterative minimum-degree heuristic algorithm to measure the topological and functional importance of a node (that is, the likelihood of being a hub or bottleneck), and the network alignment is obtained by choosing protein pairs with high alignment score by, again, a greedy, seed-and-extend approach. In L-GRAAL, node similarity is measured by considering 2-node to 4-node graphlet (connected subgraph) degree similarity and, based on node similarity, seeds are obtained using Integer Linear Programming (ILP) and Lagrangian relaxation and then extended to a network alignment using a greedy heuristic algorithm. Last, but not least, in AligNet, an overlapping clustering for every node in every network is computed. Then, all clusters pairs are aligned and scored based on sequence similarity of proteins and their neighbours. Finally, the clusters in one network are aligned with the clusters in the other network using the Hungarian algorithm, and local network alignments are first obtained as solutions to weighted bipartite hypergraph problem instances and then extended to a global network alignment.

In order to evaluate the alignments we considered the work reported in [10, 11] where several *topological coherence* and *biological coherence* measures were proposed for the comparison of protein-protein interaction network alignment methods and tools. It is shown in [11] that there is a strong correlation among the various topological coherence measures and also among the various biological coherence measures, while there is a weak correlation between the topological coherence measures and the biological coherence measures. Therefore, we have chosen one topological coherence measure and one biological coherence measure for assessing the quality of virus-host protein-protein interaction network alignments: EC, the *edge correctness* score, defined as the ratio of the interactions that are preserved by the alignment over the total number of interactions [10], and the *sequence similarity score*, a measure of functional coherence (FC), defined as the normalized sum of the sequence similarities (correlation of amino acid composition [12]) of the aligned proteins.

**Fig. 1:**
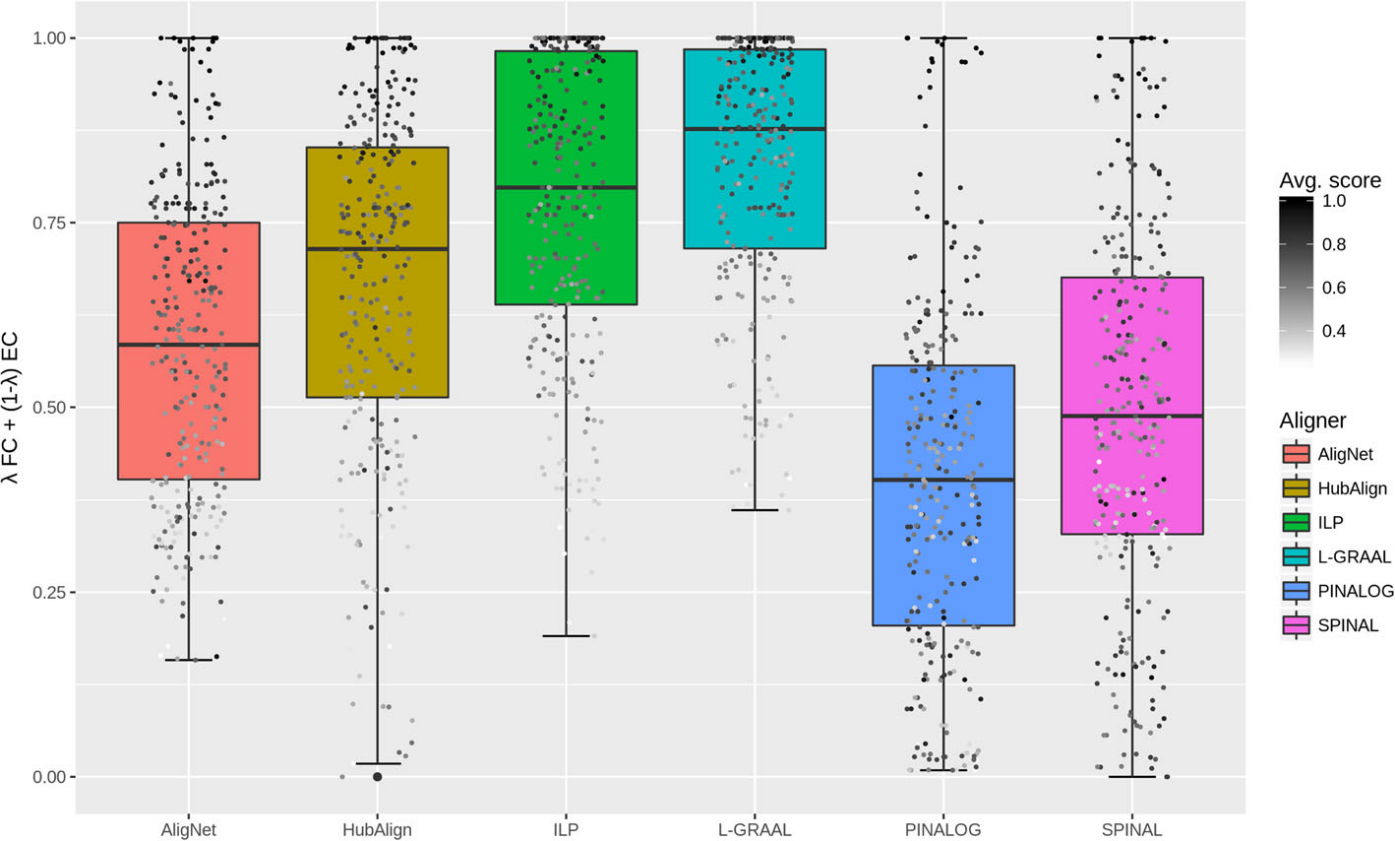
EC Scores (*λ*=0). Boxplot of EC scores for the 300 alignments of 25 virus-host protein-protein interaction networks from the STRING Viruses database, for *λ*=0. L-GRAAL and ILP obtained the highest scores

**Fig. 2:**
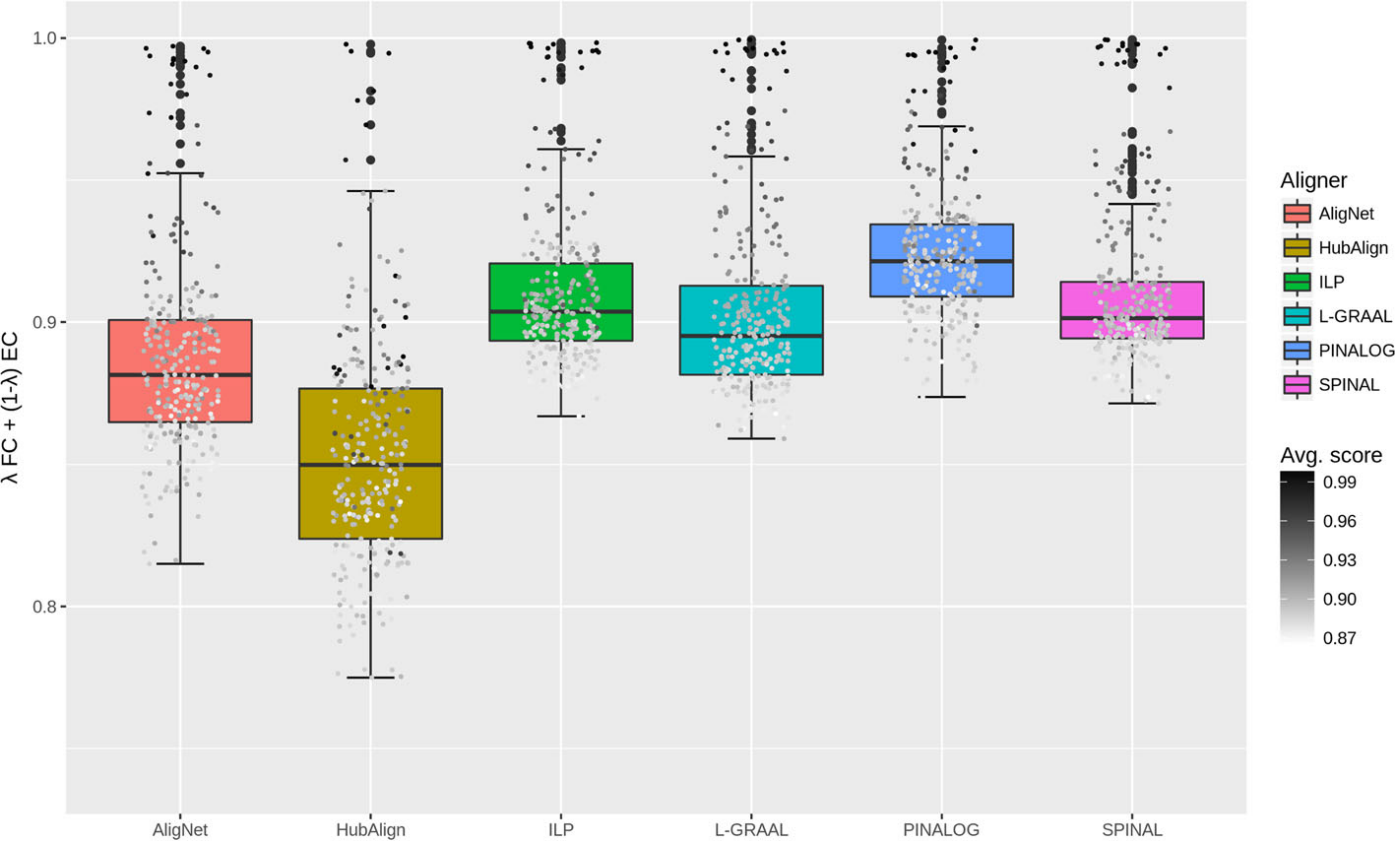
FC Scores (*λ*=1). Boxplot of FC scores for the 300 alignments of 25 virus-host protein-protein interaction networks from the STRING Viruses database, for *λ*=1. PINALOG, followed by ILP, obtained the highest scores

**Fig. 3:**
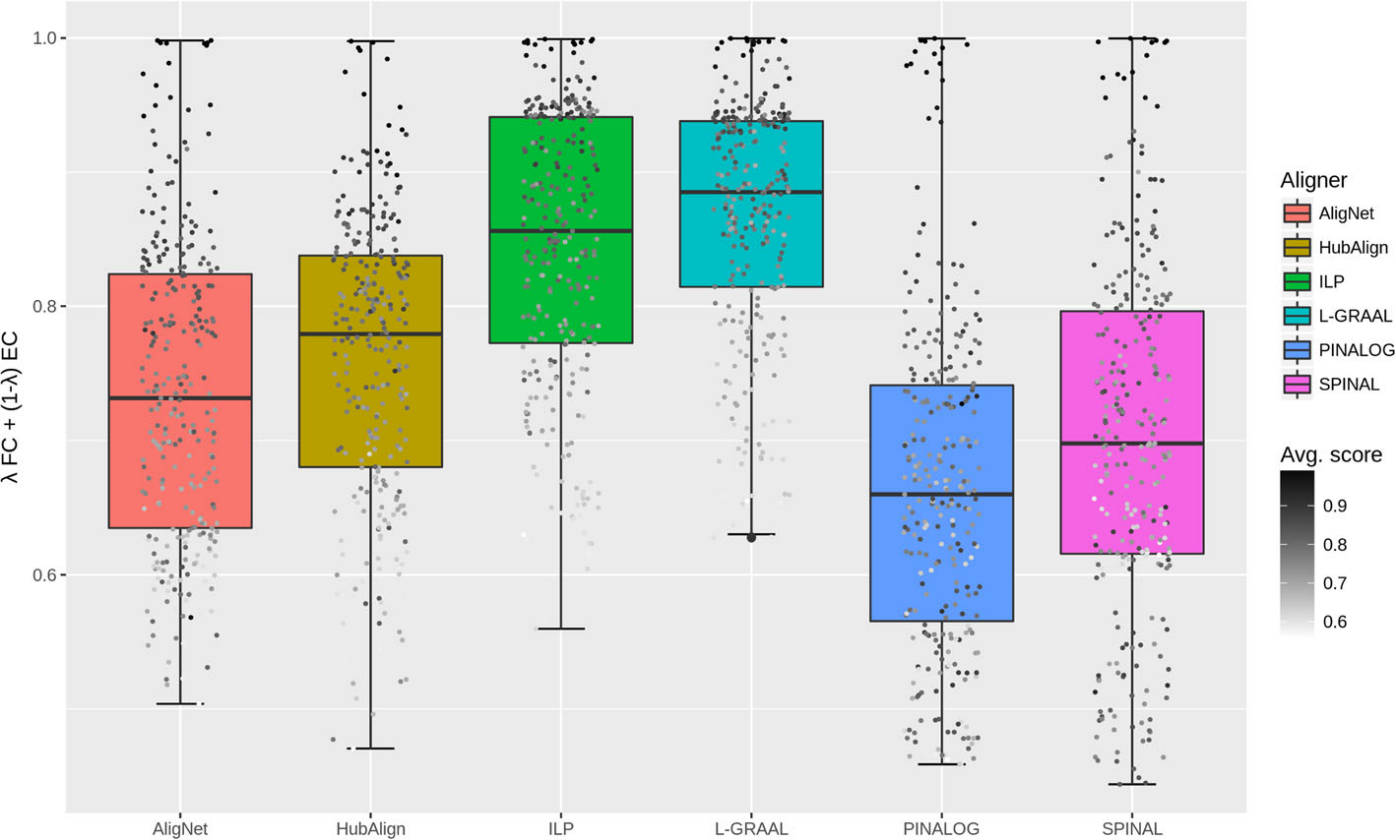
Combined EC and FC scores (*λ*=0.5). Boxplot of the mean of EC and FC scores for the 300 alignments of 25 virus-host protein-protein interaction networks from the STRING Viruses database, for *λ*=0.5. ILP and L-GRAAL obtained the highest scores

In Fig. 1 we show the boxplot of the edge correctness scores obtained for every alignment with the six alignment methods and tools considered in this study. We can observe there that L-GRAAL and our ILP method obtained the best results, with mean EC scores of 0.83 and 0.78, respectively. As far as biological coherence goes, in Fig. 2 we observe that PINALOG, being the alignment tool with the lowest EC scores, is the tool that reached the highest FC scores, with a mean FC score of 0.92, followed by our ILP method, with a mean FC score of 0.90. We can also observe that, as stated in [10, 11], some alignment methods and tools obtain either high EC scores but low FC scores, or low EC scores but high FC scores. As a measure of a balance between topological and biological coherence, we took the mean of the EC and FC scores, whose boxplot we show in Fig. 3. We can observe that, again, L-GRAAL and our ILP method obtained the best scores, followed by HubAlign and AligNet.

Table 1 shows the mean edge correctness and sequence similarity scores for all the six alignment approaches considered in this text, for the 300 pairs of virus-host protein-protein interaction networks from the STRING Viruses database described above. Moreover, Table 2 illustrates the trade-off between the conservation of interactions and the alignment of similar proteins, for a subset of 45 pairs of virus-host protein-protein interaction networks, as a function of a parameter *λ*∈[0,1] that controls the balance between protein similarity scores and protein-protein interaction weights in our model (see the “Methods” section for more details). With *λ*=0, we obtain an alignment with the highest topological coherence but with the lowest biological coherence, while *λ*=1 produces an alignment with the lowest topological coherence but with the highest biological coherence.

**Table 1:** Edge correctness score and sequence similarity score (mean values) for several protein-protein interaction network alignment methods and tools, for 300 pairs of virus-host protein-protein interaction networks from the STRING Viruses database, for *λ*=0.5. Sequence similarity scores are normalized global alignment scores

Alignment method or tool	Edge Correctness	Sequence Similarity
L-GRAAL	0.8297	0.8979
Integer Linear Programming	0.7845	0.9044
AligNet	0.5471	0.8823
PINALOG	0.3920	0.9210
HubAlign	0.6461	0.5777
SPINAL	0.5054	0.6900

**Table 2:** Edge correctness score and sequence similarity score (mean values) for 45 pairs of virus-host protein-protein interaction networks from the STRING Viruses database, for the integer linear programming formulation and different values of the *λ* parameter. The maximum sum of the edge correctness and sequence similarity scores is achieved at *λ*=0.4, followed by *λ*=0.5. Sequence similarity scores are normalized global alignment scores

*λ*	Edge	Sequence
	Correctness	Similarity
0.0	0.8655	0.7444
0.1	0.8654	0.8529
0.2	0.8648	0.8622
0.3	0.8647	0.8681
0.4	0.8612	0.8731
0.5	0.8565	0.8770
0.6	0.8493	0.8801
0.7	0.8299	0.8872
0.8	0.7820	0.8960
0.9	0.6698	0.9057
1.0	0.1054	0.9144

In order to measure the amount of variation or dispersion of the EC and FC scores used to evaluate the topological and biological coherence of the alignments, we introduced some noise to the virus-host protein-protein interaction networks by randomly adding and deleting 5% of the interactions. We computed 10,000 alignments between 100 random perturbations of the *Marburg marburgvirus* (taxid 11269) and 100 random perturbations of the *Zaire ebolavirus* (taxid 186538) virus-host protein-protein interaction networks. The mean and standard deviation of the EC and FC scores are 0.955413 and 0.012193 for the EC score and 0.991356 and 0.003269 for the FC score. That is, small perturbations of the virus-host protein-protein interaction networks produced small variations of the EC and FC scores.

While these results are based on a particular view of sequence similarity as correlation of amino acid composition, as mentioned above, it is possible to use the protein-protein interaction network alignment method with any measure of sequence similarity, including alignment-free measures such as the Euclidean distance between *k*-mer frequencies [12] and also alignment-based measures such as a normalized global alignment score. Table 3 shows the mean edge correctness and sequence similarity scores for different measures of sequence similarity (Euclidean distance between *k*-mer frequencies, for *k* between 1 and 4, and normalized global alignment score), for a subset of 45 pairs of virus-host protein-protein interaction networks with *λ*=0.5. The higher the value of *k*, the lower the mean sequence similarity score, with normalized global alignment giving the lowest score, but the mean edge correctness score is unaffected by the choice of sequence similarity measure.

**Table 3:** Edge correctness score and sequence similarity score (mean values) for 45 pairs of virus-host protein-protein interaction networks from the STRING Viruses database, for the integer linear programming formulation with *λ*=0.5 and different sequence similarity measures [12]

Sequence	Edge	Sequence
Similarity	Correctness	Similarity
1-mer	0.8540	0.8831
2-mer	0.8572	0.7229
3-mer	0.8581	0.5928
4-mer	0.8585	0.5619
Alignment	0.8465	0.3194

## Discussion

To reinforce the statement that the integer linear programming formulation of the network alignment problem provides biologically meaningful alignments of virus-host protein-protein interaction networks, we analyzed the alignments in term of agreement on virus taxonomy. Namely, we considered the taxonomy classification of the virus in every virus-host protein-protein interaction network and assumed that the highest alignment scores must be obtained when considering closely related viruses. Indeed, Table 4 shows that the best alignment (measured by the mean value of edge correctness and sequence similarity) for each of the 25 virus-host protein-protein interaction networks in Table 5, correspond to a network in the same Baltimore class [13] for 21 of the 25 best alignments. Table 5 also shows the taxonomy classification of the 25 viruses considered in our study.

**Table 4:** Best alignment for the virus-host protein-protein interaction networks for human viruses in the STRING Viruses database considered in our study. Twenty-one of the 25 networks are aligned with networks corresponding to viruses of the same Baltimore class. Sequence similarity scores are normalized global alignment scores

Virus	Virus	Edge	Sequence
Tax Id	Tax Id	Correctness	Similarity
10298	10310	0.9961	0.9950
10310	10298	0.9961	0.9950
10335	10298	0.9955	0.9877
10359	32604	0.9736	0.9638
10376	11137	1.0000	0.8885
11103	11269	1.0000	0.9057
11137	694009	1.0000	0.9933
11161	162145	0.9953	0.9951
11234	162145	1.0000	0.9934
11250	162145	0.9953	0.9969
11269	186538	1.0000	0.9964
11320	11269	1.0000	0.8824
11676	11269	1.0000	0.8836
11709	194441	1.0000	0.9078
162145	11234	1.0000	0.9934
186538	11269	1.0000	0.9964
194440	194443	1.0000	0.9980
194441	194443	1.0000	0.9984
194443	194441	1.0000	0.9984
32603	32604	0.9956	0.9933
32604	32603	0.9956	0.9933
337041	10310	0.9890	0.9050
37296	186538	1.0000	0.8867
63330	162145	0.9953	0.9949
694009	11137	1.0000	0.9933

**Table 5:** The virus-host protein-protein interaction networks for human viruses in the STRING Viruses database considered in our study

					Proteins		
Tax Id	Baltimore	Family	Genus	Species	Viral	Host	Interactions
11269	V	Filoviridae	Marburgvirus	Marburg marburgvirus	4	65	65
186538	V	Filoviridae	Ebolavirus	Zaire ebolavirus	5	66	67
194443	VI	Retroviridae	Deltaretrovirus	Primate T-lymphotropic virus 3	2	54	71
194441	VI	Retroviridae	Deltaretrovirus	Primate T-lymphotropic virus 2	2	57	74
11137	IV	Coronaviridae	Alphacoronavirus	Human coronavirus 229E	5	76	76
194440	VI	Retroviridae	Deltaretrovirus	Primate T-lymphotropic virus 1	5	67	84
694009	IV	Coronaviridae	Betacoronavirus	SARS-related coronavirus	12	87	87
337041	I	Papillomaviridae	Alphapapillomavirus	Alphapapillomavirus 9	5	80	91
11320	V	Orthomyxoviridae	Alphainfluenzavirus	Influenza A virus	11	120	144
11103	IV	Flaviviridae	Hepacivirus	Hepacivirus C	8	146	197
162145	V	Pneumoviridae	Metapneumovirus	Human metapneumovirus	5	214	215
11250	V	Pneumoviridae	Orthopneumovirus	Human orthopneumovirus	8	222	225
32604	I	Herpesviridae	Roseolovirus	Human betaherpesvirus 6B	17	201	227
32603	I	Herpesviridae	Roseolovirus	Human betaherpesvirus 6A	18	201	227
11161	V	Paramyxoviridae	Rubulavirus	Mumps rubulavirus	4	239	249
63330	V	Paramyxoviridae	Henipavirus	Hendra henipavirus	6	247	285
11234	V	Paramyxoviridae	Morbillivirus	Measles morbillivirus	8	265	289
11676	VI	Retroviridae	Lentivirus	Human immunodeficiency virus 1	10	279	301
11709	VI	Retroviridae	Lentivirus	Human immunodeficiency virus 2	5	196	315
37296	I	Herpesviridae	Rhadinovirus	Human gammaherpesvirus 8	36	300	342
10359	I	Herpesviridae	Cytomegalovirus	Human betaherpesvirus 5	40	313	380
10376	I	Herpesviridae	Lymphocryptovirus	Human gammaherpesvirus 4	35	451	547
10335	I	Herpesviridae	Varicellovirus	Human alphaherpesvirus 3	27	557	665
10310	I	Herpesviridae	Simplexvirus	Human alphaherpesvirus 2	34	607	765
10298	I	Herpesviridae	Simplexvirus	Human alphaherpesvirus 1	45	690	957

As a matter of fact, in class I (double-stranded DNA viruses), the *Alphapapillomavirus 9* network is best aligned with the *Human alphaherpesvirus 2* network; the *Human betaherpesvirus 5* network is best aligned with the *Human betaherpesvirus 6B* network; the *Human alphaherpesvirus 3* network is best aligned with the *Human alphaherpesvirus 1* network; the *Human alphaherpesvirus 1* and *Human alphaherpesvirus 2* networks are best aligned with each other; and the *Human betaherpesvirus 6A* and *Human betaherpesvirus 6B* networks are also best aligned with each other.

In class IV (positive-sense single-stranded RNA viruses), the *Human coronavirus 229E* network is best aligned with the *SARS-related coronavirus* network. In class V (negative-sense single-stranded RNA viruses), the *Influenza A virus* network is best aligned with the *Marburg marburgvirus* network; the *Human orthopneumovirus*, *Mumps rubulavirus*, and *Hendra henipavirus* networks are best aligned with the *Human metapneumovirus* network; the *Marburg marburgvirus* and *Zaire ebolavirus* networks are best aligned with each other; and the *Human metapneumovirus* and *Measles morbillivirus* networks are also best aligned with each other.

Finally, in class VI (positive-sense single-stranded RNA viruses that replicate through a DNA intermediate), the *Human immunodeficiency virus 2* network is best aligned with the *Primate T-lymphotropic virus 2* network; the *Primate T-lymphotropic virus 1* network is best aligned with the *Primate T-lymphotropic virus 3* network; and the *Primate T-lymphotropic virus 2* and *Primate T-lymphotropic virus 3* networks are also best aligned with each other.

## Conclusions

The compact integer linear programming reformulation of the protein-protein interaction network alignment problem can also be applied to similar alignment problems on graph-based representations of molecular structures, such as metabolic pathways and gene regulatory networks. The application to virus-host protein-protein interaction networks provided high scored alignments in both network topology and biological coherence, which constitutes evidence that the alignments obtained with this approach are biologically meaningful.

The alignment of virus-host protein-protein interaction networks may contribute to discover the effect of viral infection to their host. New databases with virus information have been created in the last years from the analysis of new metagenomics data [14–16]. However, one of the problems to deal with nowadays is to understand the mechanism by which viruses infect a host and to determine the viral proteins interacting with host proteins that are responsible for such an infection. New sets of Gene Ontology classes have been developed that are applicable to microbes and their hosts, improving both coverage and quality in this area of the Gene Ontology [17]. Therefore, the alignment of virus-host protein-protein interactions can reveal a useful tool to predict new functions of viral proteins related to host infection, as it has been proven to be useful for inferring new protein functions.

## Methods

The following notation will be used in this section. A protein-protein interaction network is represented by means of an undirected graph *G*=(*V*,*E*), where each node *v*∈*V* corresponds to a protein and each edge {*u*,*v*}∈*E* corresponds to an interaction between the proteins represented by the nodes *u*∈*V* and *v*∈*V*. Let *G*=(*V*,*E*) and *G*^′^=(*V*^′^,*E*^′^) be the two protein-protein interaction networks to be aligned, let *V*={*v*_1_,…,*v*_*m*_} and *V*^′^={*v*1′,…,*vn*′} be their respective sets of nodes and *A*=(*a*_*ij*_) and *B*=(*b*_*kℓ*_) be their respective adjacency matrices. Let *S*=(*s*_*ik*_) be a similarity matrix between the nodes of the two networks, with each *s*_*ik*_ the similarity score of *v*_*i*_∈*V* and *vk*′∈*V*^′^.

An alignment of *G* and *G*^′^ can be represented by a binary matrix *X*=(*x*_*ik*_), where *x*_*ik*_=1 if the *i*-th node, *v*_*i*_, of the first network is aligned with the *k*-th node, $v^{\prime }_{k}$, of the second network, and *x*_*ik*_=0 otherwise. Then, the protein-protein interaction network alignment problem has the following simple integer quadratic programming (IQP) formulation in terms of the binary variables *x*_*ik*_ [6].

**Problem IQP.** Objective:

$$\begin{aligned} &\max \lambda \sum\limits_{i=1}^{m} \sum\limits_{k=1}^{n} s_{ik}\,x_{ik} \\ &+ (1 - \lambda) \sum\limits_{i=1}^{m} \sum\limits_{k=1}^{n} \sum\limits_{j=1}^{m} \sum\limits_{\ell=1}^{n} a_{ij}\,b_{k\ell}\,x_{ik}\,x_{j\ell} \end{aligned} $$

subject to the constraints

- (Q1) *x*_*ik*_∈{0,1}, *i*=1,…,*m*, *k*=1,…,*n*
- (Q2) $\sum \limits _{k=1}^{n} x_{ik} \leqslant 1,\quad i=1,\ldots,m$
- (Q3) $\sum \limits _{i=1}^{m} x_{ik} \leqslant 1,\quad k=1,\ldots,n$

In this problem’s objective function, *λ* is a parameter, with 0≤*λ*≤1, that controls the balance between protein similarity scores and protein-protein interaction weights: only node scores are considered when *λ*=1, and only edge scores are taken into account when *λ*=0. Constraints (Q2) and (Q3) enforce that, for every *i*=1,…,*m*, at most one *x*_*ik*_ is equal to 1 (that is, that the matrix *X*=(*x*_*ik*_) defines a, possibly partial, mapping) and that, for every *k*=1,…,*n*, at most one *x*_*ik*_ is equal to 1 (that is, that the mapping defined by *X* is injective) and hence that the matrix *X* defines an alignment between the networks *G* and *G*^′^, given by {(*v*_*i*_,*vk*′)∈*V*×*V*^′^:*x*_*ik*_=1}.

The objective function above comes from the PathBLAST [18] idea that protein-protein network alignment be based on a log-probability-like criterion, with matching terms corresponding to both proteins and interactions [6]. The first sum in the objective function,

$$\sum\limits_{i=1}^{m} \sum\limits_{k=1}^{n} s_{ik}\,x_{ik}, $$

represents the global similarity of the pairs of matching proteins, while the second sum,

$$\sum\limits_{i=1}^{m} \sum\limits_{k=1}^{n} \sum\limits_{j=1}^{m} \sum\limits_{\ell=1}^{n} a_{ij}\,b_{k\ell}\,x_{ik}\,x_{j\ell}, $$

represents the number of edges that are preserved by the alignment; that is, of pairs of edges (*v*_*i*_,*v*_*j*_)∈*E* and (*vk*′,*vℓ*′)∈*E*^′^ such that *v*_*i*_ is aligned with *vk*′ and *v*_*j*_ is aligned with *vℓ*′.

This quadratic formulation has a linearization with *O*(*m*^2^*n*^2^) binary variables and constraints [7], of no practical use with current integer linear programming software tools such as IBM ILOG CPLEX Optimization Studio or Gurobi Optimizer. We present next a much more compact linearization, with only *O*(*mn*) binary variables, integer variables, and constraints, along the lines of a well-known linearization of the quadratic assignment problem [19–21].

In addition to the binary variables *x*_*ik*_ above, we introduce an integer variable *y*_*ik*_ for each *v*_*i*_∈*V* and each $v^{\prime }_{k} \in V'$. Each such new variable *y*_*ik*_ is intended to represent

$$y_{ik} = x_{ik}\sum\limits_{j=1}^{m} \sum\limits_{\ell=1}^{n} a_{ij} b_{k\ell} x_{j\ell} $$

for *i*=1,…,*m* and *k*=1,…,*n*. In this way, if *x*_*ik*_=0,*y*_*ik*_=0, and if *x*_*ik*_=1,*y*_*ik*_ is the number of edges incident to *v*_*i*_ in *G* that are preserved by the alignment.

Since

$$\begin{aligned} \sum\limits_{i=1}^{m} \sum\limits_{k=1}^{n} y_{ik} = \sum\limits_{i=1}^{m} \sum\limits_{k=1}^{n} x_{ik} \sum\limits_{j=1}^{m} \sum\limits_{\ell=1}^{n} a_{ij} b_{k\ell} x_{j\ell} \\ = \sum\limits_{i=1}^{m} \sum\limits_{k=1}^{n} \sum\limits_{j=1}^{m} \sum\limits_{\ell=1}^{n} a_{ij} b_{k\ell} x_{ik} x_{j\ell}, \end{aligned} $$

using these new variables, the objective function of Problem IQP can be rewritten as a linear function:

$$\lambda \sum\limits_{i=1}^{m} \sum\limits_{k=1}^{n} s_{ik}\,x_{ik} + (1 - \lambda) \sum\limits_{i=1}^{m} \sum\limits_{k=1}^{n} y_{ik} $$

This motivates the following linear reformulation of problem IQP:

**Problem ILP.** Objective:

$$\max \lambda \sum\limits_{i=1}^{m} \sum\limits_{k=1}^{n} s_{ik}\,x_{ik} + (1 - \lambda) \sum\limits_{i=1}^{m} \sum\limits_{k=1}^{n} y_{ik} $$

subject to the constraints

1. *x*_*ik*_∈{0,1}, *i*=1,…,*m*, *k*=1,…,*n*
2. $\sum \limits _{k=1}^{n} x_{ik} \leqslant 1,\quad i=1,\ldots,m$
3. $\sum \limits _{i=1}^{m} x_{ik} \leqslant 1,\quad k=1,\ldots,n$
4. $0 \leqslant y_{ik} \leqslant x_{ik}\sum \limits _{j=1}^{m} \sum \limits _{\ell =1}^{n} a_{ij} b_{k\ell },\quad i=1,\ldots,m,\quad k=1,\ldots,n$
5. $y_{ik} \leqslant \sum \limits _{j=1}^{m} \sum \limits _{\ell =1}^{n} a_{ij} b_{k\ell } x_{j\ell }$, *i*=1,…,*m*, *k*=1,…,*n*

This linear problem turns out to be equivalent to problem IQP, because of the following lemma:

### **Lemma 1**

A binary matrix (*x*_*ik*_) is a solution to Problem IQP if, and only if, there is an integer matrix (*y*_*ik*_) such that ((*x*_*ik*_),(*y*_*ik*_)) is a solution to Problem ILP. Moreover, when *λ*<1, if (*x*_*ik*_) is a solution to problem IQP and (*y*_*ik*_)is such that ((*x*_*ik*_),(*y*_*ik*_)) is a solution to Problem ILP, then

$$y_{ik} = x_{ik} \sum\limits_{j=1}^{m} \sum\limits_{\ell=1}^{n} a_{ij} b_{k\ell} x_{j\ell} $$

for every *i*=1,…,*m* and *k*=1,…,*n*.

### *Proof*

If *λ*=1, the second sum in the objective function of both problems vanishes and therefore (*x*_*ik*_) is a solution to problem IQP if, and only if, ((*x*_*ik*_),(*y*_*ik*_)) is a solution to problem ILP for *every* integer matrix (*y*_*ik*_).

Now, assume that *λ*<1. It is clear from the problems’ objective functions that if (*x*_*ik*_) is a solution to problem IQP, then taking for every *i*=1,…,*m* and *k*=1,…,*n*,

$$y_{ik} = x_{ik} \sum\limits_{j=1}^{m} \sum\limits_{\ell=1}^{n} a_{ij} b_{k\ell} x_{j\ell} $$

we obtain a solution ((*x*_*ik*_),(*y*_*ik*_)) to problem ILP.

Conversely, assume that ((*x*_*ik*_),(*y*_*ik*_)) is a solution to problem ILP. If $x_{i_{0}k_{0}}=0$, constraint (L4) implies that

$$y_{i_{0}k_{0}}=0=x_{i_{0}k_{0}} \sum\limits_{j=1}^{m} \sum\limits_{\ell=1}^{n} a_{i_{0}j} b_{k_{0}\ell} x_{j\ell}. $$

And if $x_{i_{0}k_{0}}=1$, by constraint (L5) we have that

$$y_{i_{0}k_{0}} \leqslant \sum\limits_{j=1}^{m} \sum\limits_{\ell=1}^{n} a_{i_{0}j} b_{k_{0}\ell} x_{j\ell}\leqslant x_{i_{0}k_{0}}\sum\limits_{j=1}^{m} \sum\limits_{\ell=1}^{n} a_{i_{0}j} b_{k_{0}\ell} $$

and this turns out to imply that, actually,

$$y_{i_{0}k_{0}}=\sum\limits_{j=1}^{m} \sum\limits_{\ell=1}^{n} a_{i_{0}j} b_{k_{0}\ell} x_{j\ell}=x_{i_{0}k_{0}}\sum\limits_{j=1}^{m} \sum\limits_{\ell=1}^{n} a_{i_{0}j} b_{k_{0}\ell} x_{j\ell}. $$

Indeed, if $x_{i_{0}k_{0}}=1$ and $y_{i_{0}k_{0}}<{\sum \nolimits }_{j=1}^{m} {\sum \nolimits }_{\ell =1}^{n} a_{i_{0}j} b_{k_{0}\ell }\allowbreak x_{j_{0}\ell }$, then the pair of matrices $\left (\left (x_{ik}\right),\left (\hat {y}_{ik}\right)\right)$ with $\hat {y}_{ik}=y_{ik}$ except for $\hat {y}_{i_{0}k_{0}}={\sum \nolimits }_{j=1}^{m} {\sum \nolimits }_{\ell =1}^{n} a_{i_{0}j} b_{k_{0}\ell } x_{j\ell }$, still satisfies constraints (L1) to (L5) and it has a larger value of the objective function in Problem ILP, which would contradict the assumption that ((*x*_*ik*_),(*y*_*ik*_)) is a solution to problem ILP.

This implies that, when *λ*<1, if ((*x*_*ik*_),(*y*_*ik*_)) is a solution to problem ILP, then

$$y_{ik}=x_{ik} \sum\limits_{j=1}^{m} \sum\limits_{\ell=1}^{n} a_{ij} b_{k\ell} x_{j\ell} $$

for every *i*=1,…,*m* and *k*=1,…,*n*. Since the constraints on (*x*_*ik*_) are the same in both problems, we conclude that (*x*_*ik*_) is a solution to problem IQP. □

Therefore, a solution ((*x*_*ik*_),(*y*_*ik*_)) of the linear reformulation ILP of the alignment problem defines an alignment between the mapped proteins in the two networks via {(*v*_*i*_,*vk*′)∈*V*×*V*^′^:*x*_*ik*_=1}.

## Supplementary information

**Additional file 1** Supplementary materials (Tables S1–S26). (PDF 67.4 kb)

## Data Availability

The datasets analysed during the current study are available in the STRING Viruses repository, http://viruses.string-db.org/download/protein.links.v10.5/9606.protein.links.v10.5.txt.gzand http://viruses.string-db.org/download/protein.sequences.v10.5.fa.gz.

## Abbreviations

EC: Edge correctness
FC: Functional coherence
ILP: Integer linear programming
IQP: Integer quadratic programming
