# Supplementary material for: Alignment of biological networks by integer linear programming: virus-host protein-protein interaction networks
Source: BMC Bioinformatics. 2020 Nov 18;21(Suppl 6):434. doi: 10.1186/s12859-020-03733-w (PMC7671827; doi:10.1186/s12859-020-03733-w)
Supplement: Supplementary file 1 — Additional file 1 Supplementary materials (Tables S1–S26). (PDF 67.4 kb) [file 12859_2020_3733_MOESM1_ESM.pdf]

Table S1: Virus-host protein-protein interaction networks for human viruses in the STRING Viruses database, ranked by the number of interactions. The latter 25 (highlighted) are those virus-host protein-protein interaction networks considered in our study

NCBI Tax Id	Family	Genus	Species	Proteins Viral	Host	Inter
10276	Poxviridae	Suipoxvirus	Swinepox virus	1	1	1
10366	Herpesviridae	Muromegalovirus	Murid betaherpesvirus 1	1	1	1
10381	Herpesviridae	Rhadinovirus	Saimiriine gammaherpesvirus 2	1	1	1
10506	Phycodnaviridae	Chlorovirus	Paramecium bursaria Chlorella virus 1	1	1	1
10561	Papillomaviridae	Xipapillomavirus	Xipapillomavirus 1	1	1	1
10567	Papillomaviridae	Iotapapillomavirus	Iotapapillomavirus 1	1	1	1
10658	Tectiviridae	Alphatectivirus	Pseudomonas virus PRD1	1	1	1
10677	Myoviridae	Muvirus	Escherichia virus Mu	1	1	1
10754	Podoviridae	Lederbergvirus	Salmonella virus P22	1	1	1
10840	Geminiviridae	Curtovirus	Beet curly top virus	1	1	1
10991	Reoviridae	Phytoreovirus	Rice dwarf virus	1	1	1
11599	Hantaviridae	Orthohantavirus	Hantaan orthohantavirus	1	1	1
11613	Tospoviridae	Orthotospovirus	Tomato spotted wilt tospovirus	1	1	1
11631	Arenaviridae	Mammarenavirus	Tacaribe mammarenavirus	1	1	1
11886	Retroviridae	Alpharetrovirus	Rous sarcoma virus	1	1	1
11976	Caliciviridae	Lagovirus	Rabbit hemorrhagic disease virus	1	1	1
12657	Herpesviridae	Percavirus	Equid gammaherpesvirus 2	1	1	1
129951	Adenoviridae	Mastadenovirus	Human mastadenovirus C	1	1	1
348604	Myoviridae	Tequatrovirus	Enterobacteria phage T4 sensu lato	1	1	1
36772	Nanoviridae	Nanovirus	Subterranean clover stunt virus	1	1	1
37128	Virgaviridae	Pomovirus	Potato mop-top virus	1	1	1
47740	Tombusviridae	Umbravirus	Groundnut rosette virus	1	1	1
694005	Coronaviridae	Betacoronavirus	Murine coronavirus	1	1	1
74501	Tobaniviridae	Torovirus	Bovine torovirus	1	1	1
10855	Microviridae	Spiromicrovirus	Spiroplasma virus SpV4	1	2	2
11588	Phenuiviridae	Phlebovirus	Rift Valley fever phlebovirus	1	2	2
11723	Retroviridae	Lentivirus	Simian immunodeficiency virus	1	2	2
11786	Retroviridae	Gammaretrovirus	Murine leukemia virus	1	2	2
1511891	Parvoviridae	Dependoparvovirus	Adeno-associated dependoparvovirus A	2	1	2
220340	Geminiviridae	Begomovirus	Bean golden yellow mosaic virus	2	2	2
36427	Reoviridae	Rotavirus	Rotavirus C	1	2	2
40051	Reoviridae	Orbivirus	Bluetongue virus	2	2	2
11176	Paramyxoviridae	Avulavirus	Avian avulavirus 1	2	3	3
12110	Picornaviridae	Aphthovirus	Foot-and-mouth disease virus	2	3	3
12216	Potyviridae	Potyvirus	Potato virus Y	1	3	3
12455	Bornaviridae		Borna disease virus	2	2	3
39720	Retroviridae	Epsilonretrovirus	Walleye dermal sarcoma virus	2	3	3
10273	Poxviridae	Leporipoxvirus	Myxoma virus	2	4	4

Table S1: Virus-host protein-protein interaction networks for human viruses in the STRING Viruses database, ranked by the number of interactions (continued)

NCBI Tax Id	Family	Genus	Species	Proteins Viral	Host	Inter
10390	Herpesviridae	Mardivirus	Gallid alphaherpesvirus 2	2	4	4
11041	Matonaviridae	Rubivirus	Rubella virus	2	4	4
11191	Paramyxoviridae	Respirovirus	Murine respirovirus	3	4	4
10623	Papillomaviridae	Kappapapillomavirus	Kappapapillomavirus 2	3	5	5
10678	Myoviridae	Punavirus	Escherichia virus P1	3	5	5
10710	Siphoviridae	Lambdavirus	Escherichia virus Lambda	4	5	5
11047	Arteriviridae	Alphaarterivirus	Alphaarterivirus equid	4	4	5
11099	Flaviviridae	Pestivirus	Bovine viral diarrhea virus 1	1	5	5
1511908	Parvoviridae	Protoparvovirus	Rodent protoparvovirus 1	2	5	5
10497	Asfarviridae	Asfivirus	African swine fever virus	4	6	6
11292	Rhabdoviridae	Lyssavirus	Rabies lyssavirus	3	3	7
12475		Deltavirus	Hepatitis delta virus	1	7	7
28875	Reoviridae	Rotavirus	Rotavirus A	4	7	7
1239565	Astroviridae	Mamastrovirus	Mamastrovirus 1	1	8	8
12461	Hepeviridae	Orthohepevirus	Orthohepevirus A	1	8	8
12618	Anelloviridae	Gyrovirus	Chicken anemia virus	1	8	8
1511900	Parvoviridae	Erythroparvovirus	Primate erythroparvovirus 1	1	8	8
12305	Bromoviridae	Cucumovirus	Cucumber mosaic virus	1	10	10
10279	Poxviridae	Molluscipoxvirus	Molluscum contagiosum virus	8	11	13
694014	Coronaviridae	Gammacoronavirus	Avian coronavirus	6	13	13
10633	Polyomaviridae	Betapolyomavirus	Macaca mulatta polyomavirus 1	2	14	14
95342	Caliciviridae	Sapovirus	Sapporo virus	2	14	14
11033	Togaviridae	Alphavirus	Semliki Forest virus	3	15	15
333926	Papillomaviridae	Gammapapillomavirus	Gammapapillomavirus 1	3	15	15
334205	Papillomaviridae	Nupapillomavirus	Nupapillomavirus 1	3	15	15
37124	Togaviridae	Alphavirus	Chikungunya virus	3	15	15
11983	Caliciviridae	Norovirus	Norwalk virus	4	17	17
337051	Papillomaviridae	Betapapillomavirus	Betapapillomavirus 1	3	17	17
11034	Togaviridae	Alphavirus	Sindbis virus	4	17	18
10258	Poxviridae	Parapoxvirus	Orf virus	9	17	19
10760	Podoviridae	Teseptimavirus	Escherichia virus T7	4	18	19
337042	Papillomaviridae	Alphapapillomavirus	Alphapapillomavirus 7	5	22	22
99182	Retroviridae	Gammaretrovirus	Murine leukemia-related retroviruses	7	19	24
11620	Arenaviridae	Mammarenavirus	Lassa mammarenavirus	1	27	27
1330524	Picornaviridae	Salivirus	Salivirus A	2	32	32
195054	Picornaviridae	Parechovirus	Parechovirus A	2	32	32
10245	Poxviridae	Orthopoxvirus	Vaccinia virus	24	30	33
11623	Arenaviridae	Mammarenavirus	Lymphocytic choriomeningitis mammarenavirus	1	35	35
38804	Poxviridae	Yatapoxvirus	Yaba monkey tumor virus	11	34	37
11089	Flaviviridae	Flavivirus	Yellow fever virus	3	41	41
12104	Picornaviridae	Cardiovirus	Cardiovirus A	6	39	43
11053	Flaviviridae	Flavivirus	Dengue virus	4	46	46
11060	Flaviviridae	Flavivirus	Dengue virus	4	46	46
11069	Flaviviridae	Flavivirus	Dengue virus	4	46	46
11072	Flaviviridae	Flavivirus	Japanese encephalitis virus	4	46	46
12092	Picornaviridae	Hepatovirus	Hepatovirus A	5	42	47

Table S1: Virus-host protein-protein interaction networks for human viruses in the STRING Viruses database, ranked by the number of interactions (continued)

NCBI Tax Id	Family	Genus	Species	Proteins Viral	Host	Inter
147711	Picornaviridae	Enterovirus	Rhinovirus A	5	42	47
11552	Orthomyxoviridae	Gammainfluenzavirus	Influenza C virus	2	50	50
334203	Papillomaviridae	Mupapillomavirus	Mupapillomavirus 1	4	45	50
11070	Flaviviridae	Flavivirus	Dengue virus	5	51	51
11082	Flaviviridae	Flavivirus	West Nile virus	7	51	52
10255	Poxviridae	Orthopoxvirus	Variola virus	23	46	55
10407	Hepadnaviridae	Orthohepadnavirus	Hepatitis B virus	4	54	60
138950	Picornaviridae	Enterovirus	Enterovirus C	7	53	62
11520	Orthomyxoviridae	Betainfluenzavirus	Influenza B virus	5	56	63
11269	Filoviridae	Marburgvirus	Marburg marburgvirus	4	65	65
186538	Filoviridae	Ebolavirus	Zaire ebolavirus	5	66	67
194443	Retroviridae	Deltaretrovirus	Primate T-lymphotropic virus 3	2	54	71
194441	Retroviridae	Deltaretrovirus	Primate T-lymphotropic virus 2	2	57	74
11137	Coronaviridae	Alphacoronavirus	Human coronavirus 229E	5	76	76
194440	Retroviridae	Deltaretrovirus	Primate T-lymphotropic virus 1	5	67	84
694009	Coronaviridae	Betacoronavirus	SARS-related coronavirus	12	87	87
337041	Papillomaviridae	Alphapapillomavirus	Alphapapillomavirus 9	5	80	91
11320	Orthomyxoviridae	Alphainfluenzavirus	Influenza A virus	11	120	144
11103	Flaviviridae	Hepacivirus	Hepacivirus C	8	146	197
162145	Pneumoviridae	Metapneumovirus	Human metapneumovirus	5	214	215
11250	Pneumoviridae	Orthopneumovirus	Human orthopneumovirus	8	222	225
32604	Herpesviridae	Roseolovirus	Human betaherpesvirus 6B	17	201	227
32603	Herpesviridae	Roseolovirus	Human betaherpesvirus 6A	18	201	227
11161	Paramyxoviridae	Rubulavirus	Mumps rubulavirus	4	239	249
63330	Paramyxoviridae	Henipavirus	Hendra henipavirus	6	247	285
11234	Paramyxoviridae	Morbillivirus	Measles morbillivirus	8	265	289
11676	Retroviridae	Lentivirus	Human immunodeficiency virus 1	10	279	301
11709	Retroviridae	Lentivirus	Human immunodeficiency virus 2	5	196	315
37296	Herpesviridae	Rhadinovirus	Human gammaherpesvirus 8	36	300	342
10359	Herpesviridae	Cytomegalovirus	Human betaherpesvirus 5	40	313	380
10376	Herpesviridae	Lymphocryptovirus	Human gammaherpesvirus 4	35	451	547
10335	Herpesviridae	Varicellovirus	Human alphaherpesvirus 3	27	557	665
10310	Herpesviridae	Simplexvirus	Human alphaherpesvirus 2	34	607	765
10298	Herpesviridae	Simplexvirus	Human alphaherpesvirus 1	45	690	957

Table S2: Marburg marburgvirus (NCBI TaxId 11269) viral proteins

STRING Viruses	UniProtKB	Protein name
L_MABVM	P31352	RNA-directed RNA polymerase L
VP24_MABVM	P35256	Membrane-associated protein VP24
VP35_MABVM	P35259	Polymerase cofactor VP35
VP40_MABVM	P35260	Matrix protein VP40

Table S3: Zaire ebolavirus (NCBI TaxId 186538) viral proteins

STRING Viruses	UniProtKB	Protein name
L_EBOZM	Q05318	RNA-directed RNA polymerase L
VP24_EBOZM	Q05322	Membrane-associated protein VP24
VP30_EBOZM	Q05323	Hexameric zinc-finger protein VP30
VP35_EBOZM	Q05127	Polymerase cofactor VP35
VP40_EBOZM	Q05128	Matrix protein VP40

Table S4: Primate T-lymphotropic virus 3 (NCBI TaxId 194443) viral proteins

STRING Viruses	UniProtKB	Protein name
PRO_0000259780	Q0R5R4	Gag polyprotein
TAX_HTL32	Q0R5R1	Protein Tax-3

Table S5: Primate T-lymphotropic virus 2 (NCBI TaxId 194441) viral proteins

STRING Viruses	UniProtKB	Protein name
PRO_0000259947	P03363	Gag-Pro-Pol polyprotein
TAX_HTLV2	P03410	Protein Tax-2

Table S6: Human coronavirus 229E (NCBI TaxId 11137) viral proteins

STRING Viruses	UniProtKB	Protein name
NCAP_CVH22	P15130	Nucleoprotein
PRO_0000037295	P0C6X1	Replicase polyprotein 1ab
PRO_0000037297	P0C6X1	Replicase polyprotein 1ab
PRO_0000037299	P0C6X1	Replicase polyprotein 1ab
SPIKE_CVH22	P15423	Spike glycoprotein

Table S7: Primate T-lymphotropic virus 1 (NCBI TaxId 194440) viral proteins

STRING Viruses	UniProtKB	Protein name
HBZ_HTL1C	P15130	Nucleoprotein
P12I_HTL1C	P0CK16	Accessory protein p12I
P30II_HTL1C	P0CK17	Accessory protein p30II
PRO_0000259942	P14078	Gag-Pro-Pol polyprotein
TAX_HTL1C	P14079	Protein Tax-1

Table S8: SARS-related coronavirus (NCBI TaxId 694009) viral proteins

STRING Viruses	UniProtKB	Protein name
AP3A_CVHSA	P59632	Protein 3a
NCAP_CVHSA	P59595	Nucleoprotein
NS7A_CVHSA	P59635	Protein 7a
ORF9B_CVHSA	P59636	Protein 9b
PRO_0000037309	P0C6X7	Replicase polyprotein 1ab
PRO_0000037310	P0C6X7	Replicase polyprotein 1ab
PRO_0000037311	P0C6X7	Replicase polyprotein 1ab
PRO_0000037312	P0C6X7	Replicase polyprotein 1ab
PRO_0000037314	P0C6X7	Replicase polyprotein 1ab
PRO_0000037315	P0C6X7	Replicase polyprotein 1ab
SPIKE_CVHSA	P59594	Spike glycoprotein
VME1_CVHSA	P59596	Membrane protein

Table S9: Alphapapillomavirus 9 (NCBI TaxId 337041) viral proteins

STRING Viruses	UniProtKB	Protein name
VE1_HPV16	P03114	Replication protein E1
VE2_HPV16	P03120	Regulatory protein E2
VE5_HPV16	P06927	Probable protein E5
VE6_HPV16	P03126	Protein E6
VE7_HPV16	P03129	Protein E7

Table S10: Influenza A virus (NCBI TaxId 11320) viral proteins

STRING Viruses	UniProtKB	Protein name
HEMA_I34A1	P03452	Hemagglutinin
M1_I34A1	P03485	Matrix protein 1
M2_I34A1	P06821	Matrix protein 2
NCAP_I34A1	P03466	Nucleoprotein
NEP_I34A1	P03508	Nuclear export protein
NRAM_I34A1	P03468	Neuraminidase
NS1_I34A1	P03496	Non-structural protein 1
PA_I34A1	P03433	Polymerase acidic protein
PB1F2_I34A1	P0C0U1	Protein PB1-F2
PB2_I34A1	P03428	Polymerase basic protein 2
RDRP_I34A1	P03431	RNA-directed RNA polymerase catalytic subunit

Table S11: Hepacivirus C (NCBI TaxId 11103) viral proteins

STRING Viruses	UniProtKB	Protein name
F_HCVH	P0C045	F protein
PRO_0000037567	P27958	Genome polyprotein
PRO_0000037570	P27958	Genome polyprotein
PRO_0000037573	P27958	Genome polyprotein
PRO_0000037574	P27958	Genome polyprotein
PRO_0000037575	P27958	Genome polyprotein
PRO_0000037576	P27958	Genome polyprotein
PRO_0000037577	P27958	Genome polyprotein

Table S12: Human metapneumovirus (NCBI TaxId 162145) viral proteins

STRING Viruses	UniProtKB	Protein name
FUS_HMPVC	Q6WB98	Fusion glycoprotein F0
M21_HMPVC	Q6WB97	Matrix M2-1
M22_HMPVC	Q6WB96	Matrix protein M2-2
MTRX_HMPVC	Q6WB99	Matrix protein
NCAP_HMPVC	Q6WBA1	Nucleoprotein

Table S13: Human orthopneumovirus (NCBI TaxId 11250) viral proteins

STRING Viruses	UniProtKB	Protein name
FUS_HRSVB	O36634	Fusion glycoprotein F0
GLYC_HRSVB	O36633	Major surface glycoprotein G
M21_HRSVB	O42050	Matrix M2-1
MATRX_HRSVB	O42049	Matrix protein
NCAP_HRSVB	O42053	Nucleoprotein
NS1_HRSVB	O42083	Non-structural protein 1
NS2_HRSVB	O42038	Non-structural protein 2
PHOSP_HRSVB	O42062	Phosphoprotein

Table S14: Human betaherpesvirus 6B (NCBI TaxId 32604) viral proteins

STRING Viruses	UniProtKB	Protein name
AN_HHV6Z	P52448	Alkaline nuclease
DNBI_HHV6Z	P52538	Major DNA-binding protein
DPOL_HHV6Z	Q9QJ32	DNA polymerase catalytic subunit
GB_HHV6Z	P36320	Envelope glycoprotein B
GCVK_HHV6Z	P52446	Probable ganciclovir kinase
GH_HHV6Z	P52543	Envelope glycoprotein H
GL_HHV6Z	P52526	Envelope glycoprotein L
ICP27_HHV6Z	P52539	mRNA export factor ICP27 homolog
IE2_HHV6Z	Q9QJ16	Immediate-early protein 2
PRIM_HHV6Z	P52540	DNA primase
U20_HHV6Z	Q9QJ46	Glycoprotein U20
UL24_HHV6Z	P52545	Protein UL24 homolog
UL31_HHV6Z	Q9WT27	Nuclear egress protein 1
UL34_HHV6Z	Q9QJ35	Nuclear egress protein 2
VGCR_HHV6Z	Q9QJ51	G-protein coupled receptor
VU51_HHV6Z	P52542	G-protein coupled receptor homolog U51
VU84_HHV6Z	P52533	Protein U84

Table S15: Human betaherpesvirus 6A (NCBI TaxId 32603) viral proteins

STRING Viruses	UniProtKB	Protein name
AN_HHV6U	P24447	Alkaline nuclease
DNBI_HHV6U	P52338	Major DNA-binding protein
DPOL_HHV6U	P28857	DNA polymerase catalytic subunit
GB_HHV6U	P28864	Envelope glycoprotein B
GCVK_HHV6U	P24446	Probable ganciclovir kinase
GH_HHV6U	P68324	Envelope glycoprotein H
GL_HHV6U	P52508	Envelope glycoprotein L
ICP27_HHV6U	P52354	mRNA export factor ICP27 homolog
IE2_HHV6U	Q77Z83	Immediate-early protein 2
PRIM_HHV6U	P52467	DNA primase
U20_HHV6U	Q69555	Glycoprotein U20
U95_HHV6U	Q89882	Uncharacterized protein U95
UL24_HHV6U	Q06092	Protein UL24 homolog
UL31_HHV6U	P28865	Nuclear egress protein 1
UL33_HHV6U	P52380	G-protein coupled receptor homolog U12
UL34_HHV6U	P52465	Nuclear egress protein 2
VU51_HHV6U	P52382	G-protein coupled receptor homolog U51
VU84_HHV6U	P52532	Protein U84

Table S16: Mumps rubulavirus (NCBI TaxId 11161) viral proteins

STRING Viruses	UniProtKB	Protein name
FUS_MUMPM	P11236	Fusion glycoprotein F0
HN_MUMPM	P11235	Hemagglutinin-neuraminidase
L_MUMPM	P30929	RNA-directed RNA polymerase L
MATRX_MUMPM	Q9JG20	Matrix protein

Table S17: Hendra henipavirus (NCBI TaxId 63330) viral proteins

STRING Viruses	UniProtKB	Protein name
FUS_HENDH	O89342	Fusion glycoprotein F0
GLYCP_HENDH	O89343	Glycoprotein G
L_HENDH	O89344	RNA-directed RNA polymerase L
MATRX_HENDH	O89341	Matrix protein
V_HENDH	O55777	Non-structural protein V
W_HENDH	P0C1C6	Protein W

Table S18: Measles morbillivirus (NCBI TaxId 11234) viral proteins

STRING Viruses	UniProtKB	Protein name
C_MEASC	Q9YZN9	Protein C
FUS_MEASC	Q786F3	Fusion glycoprotein F0
HEMA_MEASC	Q786F2	Hemagglutinin glycoprotein
L_MEASC	Q9WMB3	RNA-directed RNA polymerase L
MATRX_MEASC	Q9W850	Matrix protein
NCAP_MEASC	Q9WMB5	Nucleoprotein
PHOSP_MEASC	Q9WMB4	Phosphoprotein
V_MEASC	P0C774	Non-structural protein V

Table S19: Human immunodeficiency virus 1 (NCBI TaxId 11676) viral proteins

STRING Viruses	UniProtKB	Protein name
NEF_HV1H2	P04601	Protein Nef
PRO_0000038427	P04578	Envelope glycoprotein gp160
PRO_0000038428	P04578	Envelope glycoprotein gp160
PRO_0000038594	P04591	Gag polyprotein
PRO_0000239240	P04578	Envelope glycoprotein gp160
REV_HV1H2	P04618	Protein Rev
TAT_HV1H2	P04608	Protein Tat
VIF_HV1H2	P69723	Virion infectivity factor
VPR_HV1H2	P69726	Protein Vpr
VPU_HV1H2	P05919	Protein Vpu

Table S20: Human immunodeficiency virus 2 (NCBI TaxId 11709) viral proteins

STRING Viruses	UniProtKB	Protein name
NEF_HV2BE	P18092	Protein Nef
PRO_0000042461	P18096	Gag-Pol polyprotein
VIF_HV2BE	P18097	Virion infectivity factor
VPR_HV2BE	P18100	Protein Vpr
VPX_HV2BE	P18099	Protein Vpx

Table S21: Human gammaherpesvirus 8 (NCBI TaxId 37296) viral proteins

STRING Viruses	UniProtKB	Protein name
AN_HHV8P	Q2HR95	Shutoff alkaline exonuclease
DNBI_HHV8P	Q2HRD3	Major DNA-binding protein
DPOL_HHV8P	Q2HRD0	DNA polymerase catalytic subunit
GB_HHV8P	F5HB81	Envelope glycoprotein B
GH_HHV8P	F5HAK9	Envelope glycoprotein H
GL_HHV8P	F5HDB7	Envelope glycoprotein L
GM_HHV8P	F5HDD0	Envelope glycoprotein M
ICP27_HHV8P	Q2HR75	mRNA export factor ICP27 homolog
K1_HHV8P	Q2HRD5	Protein K1
K42_HHV8P	F5HF36	Protein K4.2
K7_HHV8P	F5HDA4	Protein K7
KBZIP_HHV8P	Q2HR82	E3 SUMO-protein ligase K-bZIP
MIR2_HHV8P	P90489	E3 ubiquitin-protein ligase MIR2
ORF36_HHV8P	F5HGH5	Viral protein kinase
ORF45_HHV8P	F5HDE4	Protein ORF45
ORF49_HHV8P	Q2HR83	Protein ORF49
ORF4_HHV8P	Q2HRD4	Complement control protein
ORF50_HHV8P	F5HCV3	Putative transcription activator ORF50
ORF73_HHV8P	Q9QR71	Protein LANA1
ORF75_HHV8P	Q9QR70	Protein ORF75
OX2V_HHV8P	P0C788	OX-2 membrane glycoprotein homolog
PAP_HHV8P	F5HID2	DNA polymerase processivity factor
PRIM_HHV8P	F5HIN0	DNA primase
RIR1_HHV8P	Q2HR67	Ribonucleoside-diphosphate reductase large subunit
UL24_HHV8P	Q2HRB2	Protein UL24 homolog
UL31_HHV8P	F5H982	Nuclear egress protein 1
UL34_HHV8P	F5HA27	Nuclear egress protein 2
UL37_HHV8P	F5HEU7	Inner tegument protein
VCYCL_HHV8P	Q77Q36	viral cyclin homolog
VFLIP_HHV8P	F5HEZ4	Viral FLICE protein
VGPCR_HHV8P	Q98146	viral G-protein coupled receptor
VIL6_HHV8P	Q2HRC7	Viral interleukin-6 homolog
VIRF1_HHV8P	F5HF68	VIRF-1
VIRF2_HHV8P	Q2HR71	Viral IRF2-like protein
VIRF3_HHV8P	F5HIC6	Viral IRF3-like protein
VIRF4_HHV8P	Q2HR73	Viral IRF4-like protein

Table S22: Human betaherpesvirus 5 (NCBI TaxId 10359) viral proteins

STRING Viruses	UniProtKB	Protein name
AN_HCMVM	F5HF49	Alkaline nuclease
DNBI_HCMVM	F5HDQ6	Major DNA-binding protein
DPOL_HCMVM	Q6SW77	DNA polymerase catalytic subunit
GB_HCMVM	F5HB53	Envelope glycoprotein B
GH_HCMVM	Q6SW67	Envelope glycoprotein H
GL_HCMVM	F5HCH8	Envelope glycoprotein L
GM_HCMVM	Q6SW43	Envelope glycoprotein M
HELI_HCMVM	F5HEN8	DNA replication helicase
ICP27_HCMVM	Q6SW73	mRNA export factor ICP27 homolog
IL10H_HCMVM	F5HC71	Viral interleukin-10 homolog
IRS1_HCMVM	Q6SW04	Protein IRS1
PP150_HCMVM	Q6SW99	Tegument protein pp150
PP65_HCMVM	Q6SW59	65 kDa phosphoprotein
PP71_HCMVM	F5HBC6	Protein pp71
PRIM_HCMVM	F5HG51	DNA primase
TRS1_HCMVM	Q6SVX2	Protein TRS1
UL117_HCMVM	F5HFA5	Protein UL117
UL142_HCMVM	F5HHH2	Membrane glycoprotein UL142
UL144_HCMVM	F5HAM0	Membrane glycoprotein UL144
UL16P_HCMVM	F5HG68	Protein UL16
UL18_HCMVM	F5HFB4	Membrane glycoprotein UL18
UL21A_HCMVM	F5HH39	Uncharacterized protein UL21A
UL33_HCMVM	Q6SW98	G-protein coupled receptor homolog UL33
UL38_HCMVM	F5HG98	Apoptosis inhibitor UL38
UL40_HCMVM	Q6SW92	Protein UL40
UL50_HCMVM	Q6SW81	Nuclear egress protein 2
UL52_HCMVM	Q6SW79	Packaging protein UL32 homolog
UL53_HCMVM	F5HFZ4	Nuclear egress protein 1
UL76_HCMVM	Q6SW66	Protein UL76
UL84_HCMVM	F5HB40	Protein UL84
UL97_HCMVM	Q6SW46	Serine/threonine protein kinase UL97
UNG_HCMVM	F5HI85	Uracil-DNA glycosylase
US03_HCMVM	F5HEU0	Membrane glycoprotein US3
US10_HCMVM	F5HFJ7	Membrane glycoprotein US10
US11_HCMVM	Q6SVZ5	Membrane glycoprotein US11
US27_HCMVM	F5HDK1	Envelope glycoprotein US27
US28_HCMVM	F5HF62	Envelope protein US28
VICA_HCMVM	F5HAY6	Viral inhibitor of caspase-8-induced apoptosis
VIE1_HCMVM	F5HCM1	55 kDa immediate-early protein 1
VIE2_HCMVM	Q6SW29	Viral transcription factor IE2

Table S23: Human gammaherpesvirus 4 (NCBI TaxId 10376) viral proteins

STRING Viruses	UniProtKB	Protein name
AN_EBVB9	P03217	Shutoff alkaline exonuclease
BARF1_EBVB9	P03228	Secreted protein BARF1
BLRF2_EBVB9	P03197	Tegument protein BLRF2
BNL2A_EBVB9	P0C739	Protein BNLF2a
BRRF1_EBVB9	P03207	Transcriptional activator BRRF1
BZLF1_EBVB9	P03206	Trans-activator protein BZLF1
DNBI_EBVB9	P03227	Major DNA-binding protein
DPOL_EBVB9	P03198	DNA polymerase catalytic subunit
EAD_EBVB9	P03191	DNA polymerase processivity factor BMRF1
EAR_EBVB9	P03182	Apoptosis regulator BHRF1
EBNA1_EBVB9	P03211	Epstein-Barr nuclear antigen 1
EBNA2_EBVB9	P12978	Epstein-Barr nuclear antigen 2
EBNA3_EBVB9	P12977	Epstein-Barr nuclear antigen 3
EBNA4_EBVB9	P03203	Epstein-Barr nuclear antigen 4
EBNA5_EBVB9	Q8AZK7	Epstein-Barr nuclear antigen leader protein
EBNA6_EBVB9	P03204	Epstein-Barr nuclear antigen 6
GB_EBVB9	P03188	Envelope glycoprotein B
GH_EBVB9	P03231	Envelope glycoprotein H
GL_EBVB9	P03212	Envelope glycoprotein L
GM_EBVB9	P03215	Envelope glycoprotein M
GP350_EBVB9	P03200	Envelope glycoprotein GP350
ICP27_EBVB9	Q04360	mRNA export factor ICP27 homolog
IL10H_EBVB9	P03180	Viral interleukin-10 homolog
KR2_EBVB9	P13288	Serine/threonine-protein kinase BGLF4
LMP1_EBVB9	P03230	Latent membrane protein 1
LMP2_EBVB9	P13285	Latent membrane protein 2
LTP_EBVB9	P03186	Large tegument protein deneddylase
MTP_EBVB9	P03179	Major tegument protein
PRIM_EBVB9	P03193	DNA primase
RIR1_EBVB9	P03190	Ribonucleoside-diphosphate reductase large subunit
RPMS1_EBVB9	Q9Q2P0	Uncharacterized protein RPMS1
RTA_EBVB9	P03209	Replication and transcription activator
UL24_EBVB9	P03232	Protein UL24 homolog
UL31_EBVB9	P0CK47	Nuclear egress protein 1
UL34_EBVB9	P03185	Nuclear egress protein 2

Table S24: Human alphaherpesvirus 3 (NCBI TaxId 10335) viral proteins

STRING Viruses	UniProtKB	Protein name
AN_VZVD	P09253	Alkaline nuclease
DNBI_VZVD	P09246	Major DNA-binding protein
DPOL_VZVD	P09252	DNA polymerase catalytic subunit
GB_VZVD	P09257	Envelope glycoprotein B
GC_VZVD	P09256	Envelope glycoprotein C
GE_VZVD	P09259	Envelope glycoprotein E
GH_VZVD	P09260	Envelope glycoprotein H
GI_VZVD	P09258	Envelope glycoprotein I
GK_VZVD	P09261	Envelope glycoprotein K
GL_VZVD	P09308	Envelope glycoprotein L
GM_VZVD	P09298	Envelope glycoprotein M
ICP22_VZVD	P09255	Transcriptional regulator ICP22 homolog
ICP27_VZVD	P09269	mRNA export factor ICP27 homolog
ICP4_VZVD	P09310	Major viral transcription factor ICP4 homolog
NP04_VZVD	P09304	Nuclear protein UL4 homolog
ORF1_VZVD	P09266	Structural protein 1
ORF57_VZVD	P09305	Uncharacterized protein 57
PRIM_VZVD	P09270	DNA primase
RIR1_VZVD	P09248	Ribonucleoside-diphosphate reductase large subunit
TEG5_VZVD	P09263	Tegument protein UL47 homolog
UL13_VZVD	P09296	Serine/threonine-protein kinase UL13 homolog
UL24_VZVD	P09288	Protein UL24 homolog
UL31_VZVD	P09283	Nuclear egress protein 1
UL34_VZVD	P09280	Nuclear egress protein 2
US03_VZVD	P09251	Serine/threonine-protein kinase US3 homolog
VP16_VZVD	P09265	Tegument protein VP16 homolog
VP22_VZVD	P09272	Tegument protein VP22

Table S25: Human alphaherpesvirus 2 (NCBI TaxId 10310) viral proteins

STRING Viruses	UniProtKB	Protein name
DNBI_HHV2H	P89452	Major DNA-binding protein
DPOL_HHV2H	P89453	DNA polymerase catalytic subunit
GB_HHV2H	P08666	Envelope glycoprotein B
GC_HHV2H	Q89730	Envelope glycoprotein C
GD_HHV2H	Q69467	Envelope glycoprotein D
GE_HHV2H	P89475	Envelope glycoprotein E
GG_HHV2H	P13290	Envelope glycoprotein G
GH_HHV2H	P89445	Envelope glycoprotein H
GI_HHV2H	P13291	Envelope glycoprotein I
GJ_HHV2H	P13293	Envelope glycoprotein J
GK_HHV2H	P22485	Envelope glycoprotein K
GL_HHV2H	P28278	Envelope glycoprotein L
GM_HHV2H	P89433	Envelope glycoprotein M
GN_HHV2H	Q86539	Envelope glycoprotein N
ICP22_HHV2H	P89474	Transcriptional regulator ICP22
ICP27_HHV2H	P28276	mRNA export factor
ICP34_HHV2H	P28283	Neurovirulence factor ICP34.5
ICP4_HHV2H	P90493	Major viral transcription factor ICP4 homolog
NP04_HHV2H	P28280	Nuclear protein UL4
P89435_HHV2H	P89435	Alkaline nuclease
PRIM_HHV2H	P89471	DNA primase
RIR1_HHV2H	P89462	Ribonucleoside-diphosphate reductase large subunit
RNB_HHV2H	P89479	Probable RNA-binding protein
TEG5_HHV2H	P89467	Tegument protein UL47
UL07_HHV2H	P89430	Cytoplasmic envelopment protein 1
UL13_HHV2H	P89436	Serine/threonine-protein kinase UL13
UL24_HHV2H	P89447	Protein UL24
UL31_HHV2H	P89454	Nuclear egress protein 1
UL34_HHV2H	P89457	Nuclear egress protein 2
US03_HHV2H	P13287	Serine/threonine-protein kinase US3
US9_HHV2H	P89477	Envelope protein US9
VP16_HHV2H	P68336	Tegument protein VP16
VP22_HHV2H	P89468	Tegument protein VP22
VP26_HHV2H	P89458	Small capsomere-interacting protein

Table S26: Human alphaherpesvirus 1 (NCBI TaxId 10298) viral proteins

STRING Viruses	UniProtKB	Protein name
AN_HHV11	P04294	Alkaline nuclease
DNBI_HHV11	P04296	Major DNA-binding protein
DPOL_HHV11	P04293	DNA polymerase catalytic subunit
GB_HHV11	P10211	Envelope glycoprotein B
GC_HHV11	P10228	Envelope glycoprotein C
GD_HHV11	Q69091	Envelope glycoprotein D
GE_HHV11	P04488	Envelope glycoprotein E
GG_HHV11	P06484	Envelope glycoprotein G
GH_HHV11	P06477	Envelope glycoprotein H
GI_HHV11	P06487	Envelope glycoprotein I
GJ_HHV11	P06480	Envelope glycoprotein J
GK_HHV11	P68331	Envelope glycoprotein K
GL_HHV11	P10185	Envelope glycoprotein L
GM_HHV11	P04288	Envelope glycoprotein M
GN_HHV11	O09800	Envelope glycoprotein N
ICP0_HHV11	P08393	E3 ubiquitin-protein ligase ICP0
ICP22_HHV11	P04485	Transcriptional regulator ICP22
ICP27_HHV11	P10238	mRNA export factor
ICP34_HHV11	P36313	Neurovirulence factor ICP34.5
ICP4_HHV11	P08392	Major viral transcription factor ICP4
KITH_HHV11	P03176	Thymidine kinase
LTP_HHV11	P10220	Large tegument protein deneddylase
NP04_HHV11	P10188	Nuclear protein UL4
OBP_HHV11	P10193	Replication origin-binding protein
PAP_HHV11	P10226	DNA polymerase processivity factor
PRIM_HHV11	P10236	DNA primase
RIR1_HHV11	P08543	Ribonucleoside-diphosphate reductase large subunit
RNB_HHV11	P04487	Accessory factor US11
SHUT_HHV11	P10225	Virion host shutoff protein
TEG3_HHV11	P04291	Tegument protein UL14
TEG5_HHV11	P10231	Tegument protein UL47
UL07_HHV11	P10191	Cytoplasmic envelopment protein 1
UL13_HHV11	P04290	Serine/threonine-protein kinase UL13
UL16_HHV11	P10200	Cytoplasmic envelopment protein 2
UL24_HHV11	P10208	Protein UL24
UL25_HHV11	P10209	Capsid vertex component 2
UL31_HHV11	P10215	Nuclear egress protein 1
UL32_HHV11	P10216	Packaging protein UL32
UL34_HHV11	P10218	Nuclear egress protein 2
UL56_HHV11	P10240	Protein UL56
US03_HHV11	P04413	Serine/threonine-protein kinase US3
US9_HHV11	P06481	Envelope protein US9
VP16_HHV11	P06492	Tegument protein VP16
VP22_HHV11	P10233	Tegument protein VP22
VP26_HHV11	P10219	Small capsomere-interacting protein
